# Commentaries on the Surgery of the War in Portugal, Spain, France and the Netherlands

**Published:** 1862-03

**Authors:** 


Commentaries on the Surgery of the War in Portugal, Spain, France and the Netherlands, from the Battle of Roliça in 1808, to that of Waterloo in 1815; with Additions relating to those in the Crimea in 1854–55, showing the improvements made during and since that period in the great Art and Science of Surgery, on all the subjects to which they relate. Revised to October, 1855, by G. J. Guthrie, F. R. S. Sixth edition. Philadelphia: J. B. Lippencott & Co., 1862.

This long and eccentric title pertains to a most valuable contribution to the literature of the Surgery of War. The nucleus of the work was originally put forth in the form of lectures published in the *London Lancet.* Subsequent changes and additions have expanded them into a duodecimo of six hundred and fourteen pages. The following criticisms and extracts will give a fair idea of the contents of the work:

Lecture 1st gives an account of the phenomena of bullet wounds, describing their course, points of entrance and exit of the missile, the progress and results of the inflammation following, and the proper treatment. The following judicious directions are worthy of note:

The treatment of simple gunshot or flesh wounds should be, under ordinary circumstances, as simple as themselves. Nothing should be applied but a piece of linen or lint, wetted with cold water; this may be retained by a strip of sticking plaster, or any other thing applicable for the purpose of keeping the injured part covered. A compress of linen, or other similar substance, moistened with cold or iced water when procurable, will be useful; and a few inches of a linen bandage may be sewed on, to prevent the compress from changing its position during sleep. When the wound becomes tender, a little oil, lard, or simple ointment may be placed over it. A roller, as a surgical application, is useless, if not injurious. At the first and second battles in Portugal, every wound had a roller applied over it; it soon became stiff, bloody, and dirty. They did no good, were for the most part cut off with scissors, and thus rendered useless. When really wanted, at a later period, they were not forthcoming. An advancing army cannot, and ought not to carry casks full of rollers into the field; and the apothecary-general had better have instead, two casks or boxes full of good wax candles; for, although every regimental surgeon ought to have four in his panniers, kept as carefully for emergencies as his capital instruments, they will require from time to time to be replaced. No roller should be more than two inches and a quarter wide, and made of good, strong, coarse linen, very much, in fact, the reverse of the rollers which have until lately been supplied to the army.

Cold or iced water may be used as long as cold is grateful to the sufferer. When it ceases to be so, it should be exchanged for warm, applied in any convenient way which modern improvements have suggested, whether by piline, gutta percha, oiled silk, etc. An evaporating poultice may be used in private life, but no poultices should be permitted in a military hospital, until the principal surgeon is satisfied they are necessary. They are generally cloaks for negligence, and sure precursors of amputation in all serious injuries of bones and joints. They are properly used to alleviate pain, stiffness, swelling, the uneasiness arising from cold, and to encourage the commencing impeded action of the vessels toward the formation of matter. As soon as the effect intended has been obtained, the poultice should be abandoned, and recourse again had to water, hot or cold, with compress and bandage. I was iu the habit of calling a poultice, when misapplied, a *cover-slut.*

The last part of the lecture speaks of bayonet wounds, which, however, are rarely met with, as troops charging with the bayonet seldom actually meet, the terror of the weapon alone being sufficient to cause one or the other party to fly. The author introduces the following incident which is sufficiently amusing, but it is difficult to see what light it throws upon the the subject of bayonet wounds:

A certain fighting regiment had the misfortune one very misty morning to have a large number of men carried off by a charge of the Polish lancers, many being also killed. The commanding officer concluded they must be all killed, for his men possessed exactly the same spirit as a part of the French Imperial guard at Waterloo. "They might be killed, but they could not by any possibility be taken prisoners." He returned them all dead accordingly. A few days afterward they reappeared, to the astonishment of everybody, having been swept off by the cavalry, and had made their escape in the retreat of the French army through the woods. The regiment from that day obtained the ludicrous name of the "Resurrection men."

Lecture two discusses traumatic erysipelas, simple inflammation, and mortification. The discussion of erysipelas is exceedingly meagre, in amount and lacks correctness in principle. The author seems unaware of the peculiar specific and poisonous character of the disease, and advises almost the same depletory treatment which would be appropriate in a simple inflammation. It is too bad that at this late day a distinguished author does not know that erysipelas is essentially a depressing disease, and that to rely on ordinary anti-phlogistics, such as purgatives and bleeding, is to the last degree injurious.

Lecture third commences by showing that when a limb is injured beyond hope of preservation, the amputation should be primary, and not secondary, and the following precepts are given on the subject:

When the wound of an extremity is of so serious a nature as to preclude all hope of saving the limb by scientific treatment, it should be amputated as soon as possible.

An amputation of the upper extremity may almost always be done from the shoulder-joint downward, without much risk to life. When necessary, the sooner it is done the better.

An amputation of any part of the lower extremity below the knee may be done forthwith, with nearly an equal chance of freedom from any immediate danger, as of the upper extremity at or near the shoulder-joint.

It is otherwise with amputations above the middle of the thigh, and up to the hip-joint. They are always attended with considerable danger.

There can be no doubt that if the knife of the surgeon could in all cases follow the ball of the enemy or the wheel of a railway carriage, and make a clean good stump, instead of leaving a contused and ragged wound, it would be greatly to the advantage of the sufferer; but as this cannot be, and an approach to it even can rarely take place, the question naturally recurs,—At what distance of time, after the receipt of the injury or accident, can the operation be performed most advantageously for the patient?

In order to answer this question distinctly, it should be considered with reference to distinct places of injury:

1st. When injuries require amputation of the arm below the shoulder-joint, or of the leg below the knee, these operations may be done at any time from the moment of infliction until after the expiration of twelve or twenty-four hours, without any detriment being sustained by the sufferer with regard to his recovery; although every one, under such circumstances, must be desirous to have the operation over. The surgeon having several equally serious cases of injury of the head or trunk brought to him at the same time as two requiring amputation of the upper extremity, may defer the latter more safely perhaps than the assistance he is also called upon to give to the other cases, the postponement of which may be attended with greater danger.

2d. This state embraces those great injuries in which the shoulder is carried away with some injury to the trunk; or the thigh is torn off at or above its middle, rendering an amputation of the upper third, or at the hip-joint, necessary. It is this or nearly this state which alone implies a doubt as to the propriety of immediate amputation, and demands further investigation. It is the state to which attention is earnestly drawn for future observation.

One of the most interesting theoretical points in this chapter is the distinct recognition by the author of two kinds of phlebitis,—the adhesive and the suppurative,—or, rather, the plastic and the aplastic; and that the plastic form almost always terminates in recovery, while the aplastic variety, with equal certainty, ends in death. He claims to have promulgated this idea forty years ago; but it is unfortunate that neither he nor other surgeons seem to have drawn from it the requisite practical inference,—that the proper treatment in these cases is to make sure, by appropriate medication, that the inflammation, if it occur, shall be of the plastic form.

For several lectures the subject of amputations is discussed in an interesting but somewhat rambling manner. The amputation at the hip-joint comes in for discussion under this head. In the Crimean war, every case of this kind died, and in the Napoleonic wars the operation was almost equally fatal, though a few cases survived. This terrible result has lead to an attempt to save life by resection of the head of the femur, in case of gunshot fractures of the head, neck or trochanteric portion of the shaft. There is no doubt that the resection should be preferred to the amputation in all cases except where the injury to the vessels ensures gangrene of the limb. The excision of the head of the femur is by no means a very dangerous operation of itself, as has often been proved by the writer of this review, in civil surgery. It is very difficult, however, for an inexperienced army surgeon, when he sees a small bullet hole in the upper part of the thigh, which has fractured the femur, with neither hæmorrhage nor great pain, to make up his mind that the patient is bound inevitably to death unless an excision is performed. Among the wounded at Fort Donelson an opportunity was afforded to observe the reluctance of surgeons to sacrifice the head of the femur after fracture, even when the same men would amputate an arm for a very bad flesh-wound, unaccompanied by fracture or injury of the vessels,—so much are persons influenced by the external appearances of things.

Some valuable tables of statistics are also given in this chapter, showing the results of capital operations of various kinds.

Lecture eighth treats in a masterly manner of hospital gangrene. We had marked several passages for extract, but have only space for the following

Conclusions.—*First.*—Hospital gangrene never occurs in isolated cases of wounds.

*Second.*—It originates only in badly-ventilated hospitals, crowded with wounded men, among and around whom cleanliness has not been too well observed.

*Third.*—It is a morbid poison, remarkably contagious, and is infectious through the medium of the atmosphere applied to the wound or ulcer.

*Fourth.*—It is possibly infectious, acting constitutionally, and producing great derangement of the system at large, although it has not been satisfactorily proved that the constitutional affection is capable of giving rise to local disease, such as an ulcer; but if an ulcer should occur from accidental or constitutional causes, it is always influenced by it when in its concentrated form.

*Fifth.*—The application of the contagious matter gives rise to a similar local disease, resembling and capable of propagating itself, and is generally followed by constitutional symptoms.

*Sixth.*—In crowded hospitals the constitutional symptoms have been sometimes observed to precede, and frequently to accompany, the appearance of the local disease.

*Seventh.*—The local disease attacks the cellular membrane principally, and is readily propagated along it, laying bare the muscular, arterial, nervous, and other structures, which soon yield to its destructive properties.

*Eighth.*—The sloughing of the arteries is rarely attended by healthy inflammation, filling up their canals by fibrin, or by that gangrenous inflammation which attends on mortification from ordinary causes, and alike obliterates their cavities. The separation of the dead parts is, therefore, accompanied by hæmorrhage, which, when from large arteries, is usually fatal.

*Ninth.*—The operation of placing a ligature on the artery at a distance, or near the seat of mischief, does not succeed, because the incision is soon attacked with the disease, unless it has been arrested in the individual part first affected, and the patient has been separated from all others suffering from it.

*Tenth.*—The local disease is to be arrested by the application of the actual or potential cautery: an iron heated red hot, or the mineral acids pure, or a solution of arsenic, or of the chloride of zinc, or of some other caustic which shall penetrate the sloughing parts, and destroy a thin layer of the unaffected part beneath them. If a sinus or sinuses have formed under the skin or between the muscles, from the extension of disease in the cellular or areolar structure, they must be laid open, and the cautery applied; for if any part affected be left untouched or undestroyed by the acid, the disease will recommence and spread from that point. The parts touched by the acids or cautery may be defended by cloths or other material, wetted with hot or cold water according to the feelings of the sufferers, and poultices of various kinds may be had recourse to, if unavoidable.

*Eleventh.*—After the diseased parts have been destroyed by the actual or potential cautery, they cease in a great measure to be contagious, and there is less chance of the disease being propagated to persons having open wounds or ulcerated surfaces. A number of wounded thus treated are less likely to disseminate the disease than one person on whom constitutional treatment alone has been tried.

*Twelfth.*—The pain and constitutional symptoms occasioned by the disease, considered as distinct from the symptoms which may be dependent on disease endemic in the country, are all relieved, and sometimes entirely removed, by the destruction of the diseased surface, which must, however, be carefully and accurately followed, to whatever distance and into whatever parts it may extend, if the salutary effect of the remedies is to be obtained.

*Thirteenth.*—On the separation of the sloughs, the ulcerated surfaces are to be treated according to the ordinary principles of surgery. They cease to eliminate the contagious principle, and do not require a specific treatment.

*Fourteenth.*—The constitutional or febrile symptoms, whenever or at whatever time they occur, are to be treated according to the nature of the fever they are supposed to represent, and especially by emetics, purgatives, and the early abstraction of blood if the fever be purely inflammatory, and by less vigorous means if the fever prevailing in the country be of a different character. Pain should be alleviated by opium, which should be freely administered.

*Fifteenth.*—The essential preventive measures are separation, cleanliness, and exposure to the open air,—the first steps toward that cure which cauterization will afterward in general accomplish.

*Sixteenth.*—If the sufferer be very young, or of a weakly habit, his strength will frequently require to be supported in the most efficient manner by a due administration of cinchona bark, wine, and a generous diet,—means often found essentially necessary after all severe attacks of debilitating diseases.

The formidable nature of this terrible disease, before the local application of caustic remedies was fully adopted, will be best understood by the following document.

Return of the number of cases of Hospital Gangrene which have appeared at the Hospital Stations in the Peninsula between 21st June and 24th December, 1813 :—

| STATIONS. | No. of cases occurred. | Dis-charged cured. | Died. | Under treat-ment. | No. operated upon. | REMARKS. |
|---|---|---|---|---|---|---|
| Santander....... | 160 | 72 | 35 | 53 | 25 | Most of these cases were sent from Vittoria. |
| Bilbao.......... | 972 | 557 | 387 | 28 | 183 | |
| Vittoria......... | 441 | 349 | 88 | 4 | 74 | Thirty-seven transferred to Santander. |
| Passages........ | 41 | 2 | 2 | | | |
| Vera........... | .... | .... | .... | .... | .... | Vera, being almost on the field of battle, had no case. |
| | 1614 | 980 | 512 | 85 | 282 | |

Lecture eighteenth gives a discussion of gunshot wounds of the skull. The examination of this topic shows that the tendency of the experience in modern wars is to discourage the operation of trephining. When a bullet has entered the brain, it drives before it comminuted fragments of skull, hair, pieces of clothing, etc. In such circumstances the ball is, in almost all cases, out of reach, and there is nothing which the trephine can do except to increase the danger. Foreign bodies near the orifice must be picked out, cold wet dressings applied, and any tendency to inflammatory fever kept down by suitable antiphlogistics; but trephining should only be made use of to enlarge the orifice, for the extraction of foreign bodies too large to be removed otherwise, or to relieve symptoms of compression. The mortality seems from some statistics to be greater in the cases trephined, than in those left to nature.

Lectures twenty and twenty-first are upon camp pneumonia and bronchitis, which are discussed with a fair degree of ability. The author gives judicious instructions to avoid bleeding in pneumonia of the typhoid type, but seems to be wholly ignorant of the use of veratrum viride, by means of which American practitioners reduce the inflammation without loss of blood: he therefore falls back upon calomel and opium. I do not know how it is in the English army, but in this country we find the use of calomel in typhoid pneumonia to be highly injudicious, unless very sparingly given. The prisoners at *Camp Douglas* have at the present time among them some two hundred cases of this form of pneumonia, resulting from their terrible exposure to snow, rain and mud at *Fort Donelson.* We find tartar emetic inadmissible for the most part in consequence of its tendency to induce a dangerous diarrhœa, and mercurials are pretty much excluded by their depressing influence. We rely therefore on a combination of veratrum viride and laudanum during the first stages, with dover's powder, and sometimes chlorate of potash as adjuvants. A little later we resort to quinine, tinct. cinchona, tinct. iron, and other tonics.

The work before us contains some remarks upon the subject of anæsthetics. High authorities in the British army were opposed to their use in the Crimea, nevertheless, they were very extensively employed in all the divisions of the English forces except one, and among all the French troops without exception. Only one death occurred from their use among the former, and not a single one among the latter. The results of military experience are in favor of anæsthetics; and for the hurried operations of the field, the prompt action and small bulk of chloroform will give it preference over other agents; but in post hospitals, where more leisure is allowed, a mixture of chloroform and ether, or ether alone, should be employed, on account of its greater safety. E. A.

